# Human Stimulator of Interferon Genes Promotes Rhinovirus C Replication in Mouse Cells In Vitro and In Vivo

**DOI:** 10.3390/v16081282

**Published:** 2024-08-10

**Authors:** Monty E. Goldstein, Maxinne A. Ignacio, Jeffrey M. Loube, Matthew R. Whorton, Margaret A. Scull

**Affiliations:** 1 Department of Cell Biology and Molecular Genetics, Maryland Pathogen Research Institute, 3134 Biosciences Research Building, University of Maryland, College Park, MD 20742, USA; 2 Vollum Institute, Oregon Health and Science University, Portland, OR 97239, USA

**Keywords:** rhinovirus C, mouse model, CDHR3, STING

## Abstract

Rhinovirus C (RV-C) infects airway epithelial cells and is an important cause of acute respiratory disease in humans. To interrogate the mechanisms of RV-C-mediated disease, animal models are essential. Towards this, RV-C infection was recently reported in wild-type (WT) mice, yet, titers were not sustained. Therefore, the requirements for RV-C infection in mice remain unclear. Notably, prior work has implicated human cadherin-related family member 3 (CDHR3) and stimulator of interferon genes (STING) as essential host factors for virus uptake and replication, respectively. Here, we report that even though human (h) and murine (m) CDHR3 orthologs have similar tissue distribution, amino acid sequence homology is limited. Further, while RV-C can replicate in mouse lung epithelial type 1 (LET1) cells and produce infectious virus, we observed a significant increase in the frequency and intensity of dsRNA-positive cells following hSTING expression. Based on these findings, we sought to assess the impact of hCDHR3 and hSTING on RV-C infection in mice in vivo. Thus, we developed hCDHR3 transgenic mice, and utilized adeno-associated virus (AAV) to deliver hSTING to the murine airways. Subsequent challenge of these mice with RV-C15 revealed significantly higher titers 24 h post-infection in mice expressing both hCDHR3 and hSTING—compared to either WT mice, or mice with hCDHR3 or hSTING alone, indicating more efficient infection. Ultimately, this mouse model can be further engineered to establish a robust in vivo model, recapitulating viral dynamics and disease.

## 1. Introduction

Responsible for over 40% of respiratory virus infections in the human population, rhinoviruses (RVs) are highly prevalent respiratory viral pathogens [1,2,3,4]. While most often regarded as etiologic agents of the common cold, RVs can infect the lower airways, resulting in bronchiolitis or pneumonia, and are often associated with virus-induced exacerbation in acute and chronic lung diseases, such as asthma and chronic obstructive pulmonary disease (COPD) [5,6,7,8]. RVs comprise three species: RV-A, -B, and -C. While RV-A and -B infections have been described for decades [9], RV-C was only identified in 2006 [10], and has since been associated with more severe disease phenotypes [11,12] and more frequent hospitalizations within the pediatric population [2,3,13,14,15,16]. Currently, no therapeutics have been approved to prevent or treat infections by any species of RV.

Since its discovery, RV-C has proven difficult to culture in the laboratory, with many studies relying on organ cultures or differentiated primary airway epithelial cells grown at air–liquid interface (ALI) [17,18]. RV-C infection in these models has allowed for the amplification of clinical RV-C specimens [19], and revealed new insights into RV-C cell tropism [20], mechanisms of replication [21], and the host response [22]. However, while these ALI cultures provide physiologically relevant systems for probing RV-C biology, they typically lack additional cell types present in the lung during infection in vivo. To understand RV-C-mediated disease and to support the development of antivirals and vaccines against RVs, animal models are critical. However, all RV species have a very narrow host range, primarily limited to humans, and entirely restricted outside of higher primates. Both major-group and minor-group RVs have been documented to infect chimpanzees (*Pan troglodytes*) in a laboratory setting, and certain minor-group RV infections were also recorded in gibbons (*Hylobates lar*) [23,24,25]. More recently, a lethal outbreak of RV-C in Uganda within a single chimpanzee troop provided the first evidence of any naturally occurring RV infections in non-human hosts [26].

Mice represent an inexpensive, genetically tractable model with a large reagent base that has been used extensively to model viral disease; however, they are not natural hosts for RV. Therefore, not surprisingly, the development of mouse models for major- and minor-group RVs has benefited from the expression of intercellular adhesion molecule 1 (ICAM-1) or virus adaptation, respectively [27,28,29,30,31]. Recently, Rajput and Han et al. reported infection of wild-type (WT) BALB/c mice with RV-C15 [32]. However, viral RNA copy numbers never rose above the initial inoculum and decreased rapidly 16 h post-infection (hpi). Thus, these results suggest a need for an alternative mouse model. Notably, prior work has identified cadherin-related family member 3 (CDHR3) as a required factor mediating RV-C entry [33] and, more recently, stimulator of interferon genes (STING) was found to be essential for RV-C replication [34].

Here, we define the ability of RV-C to replicate and produce infectious virus in mouse lung epithelial type I (LET1) cells [35] and utilize human STING (hSTING) to significantly increase viral replication in vitro. Applying these findings in vivo, we developed a novel mouse model expressing both hCDHR3 and hSTING, and we demonstrate that these mice support RV-C replication, but also highlight the need for additional optimization.

## 2. Materials and Methods

### 2.1. Plasmids

RV-A1A and RV-C15 cDNAs were previously described [17], and kindly provided by Drs. Anne Palmenberg and James Gern, respectively. The replication-defective (GAA) mutant was generated by overlapping PCR insertion around BsrGI-AflII restriction enzymes from the WT RV-C15 plasmid to replace the RNA-dependent RNA polymerase active site amino acids GDD at 2029–2031. The following four primers were used: BsrGI reverse: 5′-CTCCTTACTCATTGTTGTACAGGC-3′, GAA forward: 5′-GCATATGGTGCTGCTGTAGTAGTGTC-3′, GAA reverse: 5′-GACACGACGACATCATCACCATATGC-3′, AflII forward: 5′-GCAGCCTTAAGCAATGGAGAC-3′. The final plasmid was sequenced to confirm GDD to GAA mutagenesis. The tetracycline-inducible hSTING construct was previously described [34], and kindly provided by Dr. Stanley Lemon.

### 2.2. Tissue Culture

HeLa-H1 cells (CRL-1958, ATCC; Manassas, VA, USA) lack endogenous hCDHR3 expression and, therefore, do not support RV-C15 entry; however, they are permissive for RV-C15 replication and support the entire RV-A1A life cycle. HeLa-E8 cells [33] (kindly provided by Drs. James Gern and Yuri Bochkov) have been engineered to express hCDHR3 and support the entire life cycle of both RV-A1A and RV-C15. Both HeLa-H1 and HeLa-E8 cells, in addition to LET1 cells (NR-42941, BEI Resources; Manassas, VA), and LET1-hSTING were grown in DMEM with 10% heat-inactivated fetal bovine serum (FBS), and maintained at 37 °C with 5% CO_2_. HeLa-E8 medium was further supplemented with 5 μg/mL blasticidin, and LET1-hSTING media with 0.8 μg/mL puromycin. To induce hSTING expression in LET1-hSTING cells, 10 μg/mL doxycycline was added three days prior to transfection. 

### 2.3. Generation of Virus Stocks

RV-A1A was linearized using the XhoI restriction enzyme (#R0146S, New England Biolabs; Ipswich, MA, USA), and both RV-C15 and RV-C15-GAA were linearized using ClaI (#R0197S, New England Biolabs). The linear plasmid was purified using either a Monarch DNA Cleanup Kit (#T1030S, New England Biolabs) or a QiaQuick Gel Extraction Kit (#28704, Qiagen; Germantown, MD, USA) with MinElute columns (#28004, Qiagen). In vitro-transcribed RV RNAs were synthesized using the MEGAscript T7 Kit (#AM1337, Invitrogen; Carlsbad, CA, USA) and infectious virus stocks were then produced in HeLa-H1 cells by transfection of in vitro-transcribed RNA. For RV-A1A, 7.0 × 10^5^ HeLa-H1 cells were seeded per well into a 24-well plate, while for RV-C15, HeLa-H1 cells were grown in a T75 to 90% confluence before transfecting with JetPrime (#114-07, Polyplus; Illkirch, FR, USA), following the manufacturer’s protocol. When generating RV-C15 stocks, double the amount of RNA and JetPrime reagent were used. At four hours post-transfection, the medium was replaced with McCoy’s 5a medium (#16600082, Gibco; Waltham, MA, USA) containing 2% FBS, 1% penicillin/streptomycin (#15140-122, Gibco), 1% L-glutamine (#25030081; Gibco), and 30 mM MgCl_2_ and incubated at 34 °C. RV-C15 was collected 24 h post-transfection (hpt), while RV-A1A was collected after observing robust cell death. RV-A1A was then amplified in T75 flasks containing confluent HeLa-H1 monolayers. Final RV-A1A stocks were collected upon observing cytopathic effects (typically 48-72 hpi), clarified by centrifugation at 1000× *g* for 10 min, and titered by plaque assay. RV-C15 stocks were quantified by qPCR to determine the genome copy number.

For ultraviolet (UV) inactivation, RV-C15 was stored on ice and exposed to UV light inside a biological safety cabinet for two hours. To confirm complete inactivation, RV-A16 was inactivated in parallel and subsequently titered to ensure undetectable plaque-forming units.

Adeno-associated viruses (AAV6.2) expressing human STING (AAV-hSTING) or firefly luciferase (AAV-Fluc) were purchased from VectorBuilder Incorporated. 

### 2.4. Transfection

To assess viral replication in LET1, uninduced LET1-hSTING, doxycycline-induced LET1-hSTING (all 4.0 × 10^4^/well), and HeLa-H1 cells (6.5 × 10^4^/well), cells were seeded in 8-well chamber slides (#CCS-8, MatTek Corporation; Ashland, MA, USA) in DMEM (#25-500, Genesee Scientific; El Cajon, CA, USA) with 10% FBS and incubated overnight at 37 °C and 5% CO_2_. An amount of 500 ng of viral genomic RNA was then transfected with 1 μL of JetPrime reagent using the JetPrime protocol. Four hours post-transfection, the medium was replaced, as described in Section 2.3. Twenty-four hours post-transfection, supernatants were collected and passed through Spin-X^®^ centrifuge tube 0.45 μm filters (#8162; Corning; Corning, NY, USA) by centrifugation. All cells were then fixed (see Section 2.9). For reinfection assays, filtered supernatants were directly added to 7.5 × 10^4^ HeLa-E8 cells seeded the day prior in an 8-well chamber slide and incubated for 24 h at 37 °C.

### 2.5. Plaque Assay

RV-A1A stocks were titrated by plaque assay using HeLa-H1 cells in 24-well plates and a published protocol with modifications [36]. Briefly, 90% confluent HeLa-H1 monolayers were inoculated with ten-fold serial dilutions of the viral stock in McCoy’s 5a medium with 2% FBS and 30 mM MgCl_2_. After a 1 h incubation at room temperature, the inoculum was exchanged for a semi-solid overlay composed of 0.6% bacteriological agar (#A5306; Sigma-Aldrich; Burlington, MA, USA) in DMEM-F12 (#12500062; Gibco), supplemented with 1% FBS, 1% penicillin/streptomycin, 1% L-glutamine, and 30 mM MgCl_2_. The plate was then incubated at 34 °C for 72 h before fixing the cells with 4% formaldehyde (#47608; Sigma-Aldrich; diluted in a 0.15 M saline solution). The fixing solution was removed together with the overlay after a 24-h incubation at room temperature, and plaques were visualized by staining the monolayer with 0.1% Crystal Violet (#C0775; Sigma-Aldrich) diluted in a 20% ethanol solution.

### 2.6. RV Genome Quantification by qPCR

To quantify viable RV genomes in RV stocks, each 100 μL sample was first treated with 1 u RNase A (#EN053; Thermo Scientific; Waltham, MA, USA) and 1 u DNase I (#18047019; Invitrogen) for 1 h at 37 °C, and then inactivated at 70 °C for 15 min. Viral RNA was then extracted using a QIAamp Viral RNA Mini Kit (#52904; Qiagen). For RNA extraction from whole tissues, tissues were homogenized using a TissueLyser (Qiagen) using 5 mm stainless steel beads (#69989; Qiagen) for 15 min at 50 oscillations/sec. Total RNA was then extracted using an RNeasy Universal Kit (#73404; Qiagen). 

To reverse transcribe extracted RNA into cDNA, 100 ng of total RNA was used in a reverse transcription reaction (High Capacity cDNA Reverse Transcriptase Kit; #4368814; Applied Biosystems; Waltham, MA, USA), with random hexamer primers, as per the manufacturer’s protocol. For cDNA synthesis after viral RNA extraction from supernatants, a fixed 10 μL volume was used. 

qPCR was done using 3 μL of cDNA, 10 pM HRVTF63 forward (5′–ACMGTGTYCTAGCCTGCGTGGC–3′) and HRVTR reverse (5′–GAAACACGGACACCCAAAGTGT–3′) primers, 10 pM HRVTF probe (5′–FAM/TCCTCCGGCCCCTGAAT/ BHQ1–3′), and LuminoCT Taqman Master Mix (#L6669; Sigma-Aldrich) according to the manufacturer’s protocol. For absolute quantification, a standard curve was delineated using cDNA generated from ten-fold serial dilutions of in vitro-transcribed pRV-C15 or pRV-A1A. This standard curve of RNA was used to calculate an exponential regression line when plotted on a semi-log scale. This formula could then be used to input the cycle threshold (Ct) value of interest to calculate the genome copy number.

A 50 μL pre-amplification supermix (#1725160; Bio-Rad Laboratories; Hercules, CA, USA) reaction was performed prior to running a Taqman Probe qPCR reaction to amplify RV signal from mouse lung tissue. Following the manufacturer’s protocol, 2.5 μL of forward and reverse primers at 100 μM were diluted with 495 μL of water. Then, 25 μL of supermix was combined with 5.0 μL of the diluted forward and reverse primer mixture, 19 μL of water, and 1 μL of the specific cDNA to reach a 50 μL total volume reaction.

### 2.7. Host Gene Expression Quantification by qPCR

Total RNA was extracted, and cDNA was prepared as detailed above. Relative gene expression was quantified using the two-step QuantiFast SYBR Green Kit (#204054; Qiagen) and a Roche LC480 thermocycler according to the manufacturer’s protocol. Primers were as follows: mCDHR3: 5′-AGGACCGAAAGCATTCGATTT-3′ and 5′-AGGCTCGTTCACATCTGTCAC-3′; murine glyceraldehyde3 3-phosphate dehydrogenase (GAPDH; endogenous control): 5′-AGGTCGGTGTGAACGGATTTG-3′ and 5′-GGGGTCGTTGATGGCAACA-3′. Human CDHR3 primers were 5′-TTACCTGCTACAGGCAATGTG-3′ and 5′-CCTGACAGCCAATTCACCCTAA-3′. 

### 2.8. SDS-PAGE and Western Blot

Cell lysates were collected in RIPA buffer containing protease inhibitors (#N653-500 mL, VWR; Radnor, PA, USA) and total protein was quantified using a Pierce BCA assay (#23227, Thermo Scientific). Protein lysates (25 µg) were denatured by heating (98 °C, 2 min) and separated on a Novex^TM^ WedgeWell 4–20% Tris-Glycine gel (#XP04202BOX; Invitrogen) under reducing conditions. Proteins were then transferred to PVDF membranes (GE Healthcare Life Sciences; Chicago, IL, USA or using eBlot L1 (GenScript; Piscataway, NJ, USA)). 

Membranes were blocked in 5% (wt/vol) skim milk powder diluted in tris-buffered saline with 0.1% Tween-20 (TBS-T; #BP337-100, Fisher Scientific; Waltham, MA, USA) for 1 h at room temperature with rocking. STING was detected endogenously (#19851-1-AP, 1:1,000; Proteintech; Rosemont, IL, USA) or by HA-tag (#26183 1:1000, Invitrogen). Following incubation overnight at 4 °C, membranes were washed 3× for 15 min each with TBS-T before incubation with HRP-conjugated donkey anti-rabbit IgG (1:10,000; #A16035, Invitrogen) or HRP-conjugated mouse IgGκ light chain binding protein (sc-516102, 1:10,000; Santa Cruz Biotechnology; Dallas, TX, USA). Anti-β-Actin-HRP conjugated antibody (#A3854, Sigma-Aldrich) was diluted 1:35,000 in 5% TBS-T and incubated at room temperature for one hour. Western blots were visualized with SuperSignal West dura (#34075, Thermo Scientific), or femto (#35095, Thermo Scientific) reagent and imaged on either FujiFilm LAS-3000 (Tokyo; Japan) or Thermo Fisher iBright FL15000 imagers.

### 2.9. Immunofluorescence

Twenty-four hours post-transfection or -infection, cells were fixed with either 4% paraformaldehyde (Electron Microscopy Sciences) for 15 min (LET1 cells), or ice-cold methanol for 10 min (HeLa cells). Following fixation, cells were washed 2× in phosphate-buffered saline (PBS) and 3× in PBS with 1 mM CaCl_2_ and 1 mM MgCl_2_ (PBS++), then permeabilized with 0.2% Triton X-100 (#TX15681, Sigma-Aldrich) in PBS for 15 min. After permeabilization, cells were blocked in 10% donkey serum in PBS for 15 min in a humidified chamber at room temperature. J2 antibody (#10010200, Scicons; Susteren, The Netherlands) was diluted 1:1000 in 1% bovine serum albumin (BSA)/PBS and incubated with samples overnight at 4 °C. Cells were then washed with PBS prior to secondary antibody staining. Secondary antibody AlexaFluor555 anti-mouse IgG2a at a 1:200 dilution was applied for 1 h at room temperature in a humidified chamber. Cells were washed with PBS and incubated with Hoechst (1:1000, 10 min; Thermo Scientific). Images were acquired with a Zeiss Axio Observer 3 Inverted fluorescence microscope and Zeiss Axiocam 503 mono camera (White Plains, NY, USA). 

We used CellProfiler [37] to quantify the percentage of dsRNA(+) LET1 cells, as well as fluorescence intensity within infected cells. Individual channels of nuclei, dsRNA (J2), and long-exposure auto-fluorescent (488 nm) images were loaded into the CellProfiler pipeline. These images were taken at 63× magnification from four predetermined locations within the transfected culture. CellProfiler defines the cell borders of the cells using the auto-fluorescent signal. Next, nuclei were isolated by CellProfiler and were superimposed on the defined cell boundaries. Only those cells which contained nuclei were deemed proper cells and subsequently analyzed. Next, J2 signal was isolated to individual objects or units of similarly intense regions of signal, and overlaid onto proper nucleated cells. Integrated J2 intensity within a cell was calculated as the summation of all J2 object intensities. Cells were scored as infected when the integrated J2 intensity within a cell was over 100. 

### 2.10. In Situ Hybridization (ISH)

Twenty-four hours post-transfection or -infection, cells were fixed with 4% paraformaldehyde for 15 min, washed with PBS, and then quenched with 25 mM NH_4_Cl (#A9434-500G, Sigma Aldrich) for 10 min. Slides were washed 3× with PBS before permeabilization with 0.25% Triton X-100 in PBS. Slides were incubated with a prehybridization mix containing 3% BSA and 4X sodium citrate buffer (#C8532-100g, Thermo Scientific) for 20 min at 55 °C. ISH probe 5′-DigN/GGA+YGG+RACC+RACTACTTTGG+RTGTCC-3′DigN (RV), or 5′-DigN/CTCATTGTAGAAGGTGTGGTGCCA-3′DigN (β-Actin), was diluted to 2.5 nM in 1 mL of pre-warmed hybridization buffer of 10% dextran sulfate in 4× sodium citrate buffer. ISH probes were hybridized at 55 °C for 1 h. A series of washes using buffers pre-warmed to 55 °C was performed on a rocker: 3 washes with 4× sodium citrate buffer with 0.1% Tween-20 for 5 min each, 2 washes with 2× sodium citrate buffer for 5 min each, 1 wash with 1× sodium citrate buffer for 5 min. Lastly, a room temperature wash with diethyl pyrocarbonate (DEPC; #D5758, Sigma-Aldrich) PBS was performed. Endogenous peroxides were quenched with 3% hydrogen peroxide (#216763, Sigma-Aldrich) for 20 min in a light-protected environment. Slides were washed with DEPC PBS for 5 min before blocking with 4% BSA, 3% donkey serum (#017-000-121, Jackson Laboratories; Bar Harbor, ME), 3% goat serum (#005-000-121, Jackson Laboratories), 0.1% Triton X-100 in DEPC PBS for 30 min. Goat anti-digoxigenin antibody (#MB-7000, Vector Laboratories; Newark, CA) was diluted 1:500 in a dilution buffer of 4% BSA and 0.1% Triton X-100 in DEPC PBS and applied to the slides overnight at 4 °C. Slides were washed twice with DEPC PBS with 0.1% Tween-20 (DEPC PBS-T) for 10 min each and twice again with DEPC PBS-T for 5 min each. Rabbit anti-goat biotinylated secondary antibody (#BA-1000, Vector Laboratories) was diluted 1:500 in dilution buffer and incubated at room temperature for 30 min. Slides were again washed 4× with DEPC PBS for 5 min on a rocker. The signal was amplified using Streptavidin-conjugated horseradish peroxidase (#RABHRP3, Sigma-Aldrich) diluted 1:300 in dilution buffer for 30 min. Slides were washed 3× with DEPC PBS for 5 min each on a rocker and color was revealed using a NovaRed Substrate Kit (#SK-4800, Vector Laboratories) for 5 min in a light-protected environment. The color reaction was stopped by washing twice with DEPC PBS for 5 min. Slides were washed 6× for 3 min each with DEPC H_2_O. To prepare for imaging, slides were dehydrated using the following series of washes: 95% ethanol with 1% acetic acid (#A38-212, Fisher Scientific), 95% ethanol, 100% ethanol, 100% ethanol, 50% ethanol with 50% xylenes (#X3P-1GAL, Fisher Scientific), 100% xylenes, 100% xylenes, and a final 100% xylene wash for 5 min. VectaShield and coverslip addition were performed in the same manner as was done for IHC.

### 2.11. Immunohistochemistry

Paraffin-embedded, BALB/c mouse lung tissue sections (Zyagen; San Diego, CA, USA) or C57BL/6 (Experimental Pathology Research Laboratory at New York University, New York, NY) on glass slides were used in immunohistochemistry assays. Deparaffinization was performed using the following series of washes: 2× 100% xylene for 5 min each, 2× 100% ethanol for 3 min each, 2× 90% ethanol for 3 min each, 1× 70% ethanol for 3 min, and 1× H_2_O for 10 min. For dsRNA detection, prior to the blocking step, heat-induced epitope retrieval was performed by heating the slides in sodium citrate buffer (10 mM sodium citrate, 0.05% Tween-20 in dH_2_O) at 95 °C for 15 min. Slides were then incubated with 3% BSA in PBS++ for 1 h at room temperature in a humidified chamber to prevent nonspecific binding. Blocking solution was removed and antibodies were freshly diluted in 1% BSA as follows: rabbit anti-mCDHR3 (#orb182906, Biorbyt; Cambridge, UK) 1:3200 and isotype control IgG (#GTX35035, GeneTex Inc.; Irvine, CA, USA), 1:32,000; rabbit anti-hCDHR3 (#HPA011218, Sigma) 1:1000 and isotype control IgG (#GTX35035, GeneTex), 1:10,000; and mouse anti-acetylated alpha tubulin (#Ab24610, Abcam; Waltham, MA, USA), 1:2000. J2 (#100010200, Scicons) or IgG2a isotype control (#02-6200, Thermo Scientific) was diluted 1:500 in 1% BSA. Antibodies were added to the lung tissue sections and incubated at room temperature in a humidified chamber overnight. The slides were then washed 3× for 5 min with PBS++ prior to the addition of secondary antibodies, AlexaFluor647 anti-rabbit IgG and AlexaFluor555 anti-mouse IgG diluted 1:500 in 1% BSA, for 1 h at room temperature in a humidified chamber. For dsRNA detection, AlexaFluor555 anti-mouse IgG2a was diluted 1:200 in 1% BSA. Slides were washed once for 5 min with PBS++ before DAPI (#62248, Thermo Scientific) diluted to a final concentration of 1:1000, or Hoechst 1:1000 was added to the slides for 10 min. Slides were washed two additional times with PBS++ and tissue sections were mounted with VectaShield mounting media prior to imaging on an Axio Observer 3 inverted fluorescence microscope (Zeiss) equipped with an AxioCam 503 mono camera and Zeiss Zen 2.3 lite software for image acquisition and processing. For dsRNA staining, a VectaShield containing DAPI (#H-1200, Vector Laboratories) was used and, thus, no additional nuclear staining was required.

### 2.12. Generation of Transgenic Mice

A bacterial artificial chromosome (BAC) containing CDHR3 with point mutation C529Y (rs6967330, TGT->TAT), CTD-2262G10, was obtained from the human RPCI-11 BAC library. The appropriate modification was introduced into the BAC using bacterial recombination procedures. The C529Y variant was inserted seamlessly using annealed oligos and negative selection via an rpsL-kanaR cassette. After purification of the modified BAC, its quality was checked using pulsed-field gel electrophoresis. Important regions of the gene of interest (such as exons) as well as the introduced modification were confirmed by sequencing. This strategy allowed the generation of a transgenic mouse line expressing the human CDHR3 gene carrying the C529Y variant under the control of the human CDHR3 promoter. CDHR3 exon 1 contained the translation initiation codon. The engineered CTD-2262G10 BAC could then be used for pronuclear injection (PNI) into fertilized C57BL/6NTac oocytes.

Human CDHR3 transgenic founder mice were made by PNI with random genome integration. After administration of hormones, superovulated C57BL/6NTac females were mated with C57BL/6NTac males. One cell-stage embryos were isolated from the oviducts at 0.5 days post-conception (dpc). For microinjection, the one cell-stage embryos were placed in a drop of hCZB medium under mineral oil. A microinjection pipette with an approximate internal diameter of 0.5 μm at the tip was used to inject the DNA solution into each embryo. After recovery, 25–35 injected one cell-stage embryos were transferred to one of the oviducts of 0.5 dpc pseudopregnant NMRI females. 

The integration of the transgene was evaluated by PCR assays using oligo sequences specific to the transgene. Primer sets (Table 1) were used to confirm the presence of the transgene at the 5′ end, human exon 1, human exon 6, human exon 12, 3′-UTR, and 3′ end. Each reaction contained 5 μL 0X PCR Buffer (Invitrogen), 2 μL MgCl_2_ (50 mM), 1 μL dNTPs (10 mM), 1 μL forward primer (5 μM), 1 μL reverse primer (5 μM), 1 μL control forward primer (5 μM), 1 μL control reverse primer (5 μM), 0.2 μL Taq Polymerase (5 U/μL, Invitrogen), 38.50 μL H_2_O, and 2 μL DNA. PCR was performed using the following steps: (1) 95 °C for 5 min, (2) 35 cycles of 95 °C for 30 s, 60 °C for 30 s, and 72 °C for 1 min, (3) 72 °C for 10 min.

A total of six founder mice were produced, but only four founder animals were fully validated to express the full-length transgene. One additional founder was found to be sterile, yielding three hCDHR3 transgenic founder lines to evaluate. Founder mice were then bred with C57Bl/6NTac mice for one generation to achieve offspring heterozygous for hCDHR3 transgene expression. Mice were then interbred and mice homozygous for the transgene were identified by Taqman qPCR (Table 2) after one generation. All transgenic mice were sequenced and confirmed by Transnetyx, Inc (Cordova, TN, USA). 

### 2.13. Intranasal Inoculation

Mice were anesthetized with a cocktail of 80 mg/kg of ketamine and 5.0 mg/kg xylazine diluted in sterile water and delivered via intraperitoneal (IP) injection. Upon complete sedation, a 50 µL inoculum containing either 10^11^ genome copies of adeno-associated virus 6.2 (AAV), or 1.4 × 10^10^ genome copies of RV-C15 (or UV-inactivated RV-C15), diluted in sterile PBS, was delivered across both nares. Animals were subsequently monitored to ensure recovery from anesthesia. 

### 2.14. Health Scoring and Euthanasia

All mice enrolled in an infection experiment were assessed for clinical signs of disease and scored as follows: For grooming: 0, smooth coat and bright eyes; 1, slight lack of grooming, i.e., scruffy or hunched at rest; and 2, rough coat, hunched at rest and/or crusty or closed eyes. For behavior: 0, normal activity; 1, minor changes in behavior such as slight lethargy; 2, reduced mobility, inactivity, labored breathing, and complete lethargy; and 3, immobile even after stimulation. Each animal was then weighed and the percent of the original (T = 0) weight was calculated. A weight score of 0 was used if weight was maintained or increased; a score of 1 was used if there was <10% weight loss, or 2 if there was a 10-20% weight loss, while a score of 3 was used if the animal dropped >20% of its weight. Mice were humanely euthanized using CO_2_ if they reached a total daily score of 6 or greater, or if they lost >20% of their starting body weight. 

### 2.15. Tissue Collection 

All tissues to be used in qPCR assays were placed in 500 µL of RNAlater (#AM7021, Invitrogen) upon collection and stored at 4 °C until subsequent analysis (see Section 2.6 and Section 2.7). To prepare mouse lungs for histological assays, both the diaphragm and ribcage were removed ahead of inflation with 10% formalin delivered through a blunt-end needle inserted into the trachea. After inflation, the trachea, lungs, and heart were removed from the chest cavity, placed in an ample volume of 10% formalin, and stored at room temperature overnight. The next day, the heart was removed, and the lungs were transferred to PBS before paraffin-embedding and sectioning at the Experimental Pathology Research Laboratory at New York University. 

To prepare tissues for luminescence imaging, lungs were first perfused with 20 mL of PBS using an 18-gauge needle inserted into the right ventricle. Lungs were then processed as above for formalin fixation, using 750 μL of 30 mg/mL luciferin (#122799, PerkinElmer; Shelton, CT) in place of formalin and then removed to a black 6-well plate (#07-202-461, Corning). During necropsy, nasal turbinates were also collected and placed in a separate black 6-well plate. Both lungs and nasal turbinates were soaked in 2 mL of 30 mg/mL luciferin for 15 min prior to measuring on an IVIS^®^ Spectrum In Vivo Imaging System with Living Image ® Version 4.7.4 software. Quantification of the luminescence signal was performed using Living Image 4.7.4.

## 3. Results

### 3.1. Murine and Human CDHR3 Orthologs Exhibit Similar Tissue Expression Patterns, but Lack Complete Homology

**Figure 1:**
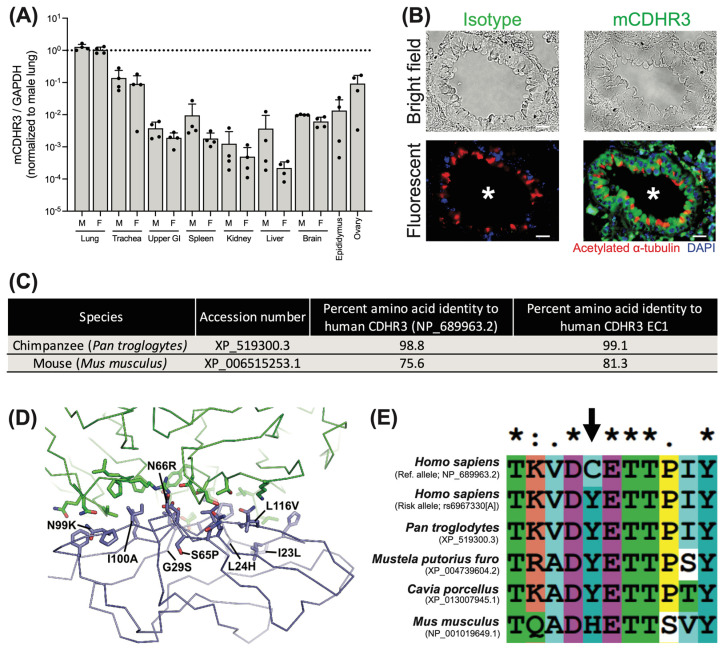
Murine CDHR3 is localized to similar tissues to human CDHR3, yet differs in several key aspects. (**A**) qPCR of murine CDHR3 expression relative to murine GAPDH by tissue across *n* = 4 male (M) and *n* = 4 female (F) mice. Mean +/− standard deviation. (**B**) Immunohistochemical detection of CDHR3 (green) in a BALB/c mouse lung tissue. Nuclei (DAPI; blue) and cilia (acetylated alpha tubulin; red). White asterisk indicates airway lumen. Scale bar = 20 µm. (**C**) Table detailing CDHR3 amino acid conservation in chimpanzees and mice. (**D**) The cryo-EM structure of RV-C15 (green) in complex with the human CDHR3 EC1 (blue) is shown as a Cα trace. The three RV-C15 capsid proteins that interact with EC1 (VP1, VP2, and VP3) are all shown as the same color. The side chains that comprise the RV-C15-EC1 interface are represented as sticks. Residues that differ from the mouse ortholog are labeled. (**E**) Homology of CDHR3 at position 529 (indicated by the black arrow), performed using ClustalX2.

RV-C is difficult to culture in standard cell lines [17,18] and CDHR3 expression in HeLa cells was subsequently shown to enable RV-C particle uptake, indicating CDHR3 acts as a receptor for this virus [33]. Thus, we initially sought to identify differences in CDHR3 expression across tissues between human and murine orthologs of CDHR3. To do so, we obtained total RNA from C57BL/6 mouse tissues, and assayed relative expression of CDHR3 by qPCR. In accordance with prior profiling of CDHR3 in human bronchial and murine tracheal epithelial tissues [38,39], we observed elevated CDHR3 expression in the mouse lung and trachea, in addition to the ovaries of female mice as compared to other tissues (Figure 1A). 

Further, immunohistochemical staining of mouse lung sections localized mCDHR3 to the ciliated epithelium, as determined by co-staining with acetylated α-tubulin (Figure 1B). These data indicate that human and mouse CDHR3 orthologs are expressed in similar tissues and cell types; however, sequence alignments revealed they are not entirely conserved at the amino acid level (Figure 1C). Specifically, the mCDHR3 protein is only 75.6% identical to hCDHR3, with 81.3% identity within the extracellular domain 1 (EC1), the region that interacts with RV-C [40,41]. This is in contrast to the near complete sequence identity of chimpanzee CDHR3, where natural RV-C infection has been documented [26]. 

An analysis of the cryo-EM structure of human CDHR3 EC1 in complex with RV-C15 shows that the capsid proteins VP1, VP2, and VP3 of RV-C15 interact with 22 residues of the EC1 (Figure 1D) [41]. Among these residues, there are eight that differ between human and mouse CDHR3. Two of these residues in human CDHR3, N66 and I100, form direct interactions with RV-C15 (G203 and T204 of VP2 for N66, and V73, P232, R234, and P235 of VP3 for I100), which would be lost in the mouse protein when these are substituted to arginine and alanine, respectively. The other six residues that differ are on the periphery of the interface and/or are substituted to relatively conserved residues; however, it is still possible that these substitutions could contribute additional negative effects on virus receptor binding by altering the shape and charge distribution of the binding interface.

Beyond the direct impacts of EC1 residues on RV-C binding, residue 529 is known to influence the surface expression of hCDHR3 [13,42], and could, therefore, impact entry into mouse cells. Notably, mCDHR3 position 529 contains a histidine residue that is distinct from RV-C-susceptible species, and may further impact cell surface expression and availability (Figure 1E). While further mutagenesis efforts are needed to measure the contributions of each mutation individually and in combination, these data indicate potential differences in cell surface expression and the RV-C—EC1 interface that may have deleterious effects on viral particle uptake into mouse cells. 

### 3.2. Murine Cells Are Permissive for RV-C, and Human STING Significantly Increases RV-C Replication

**Figure 2:**
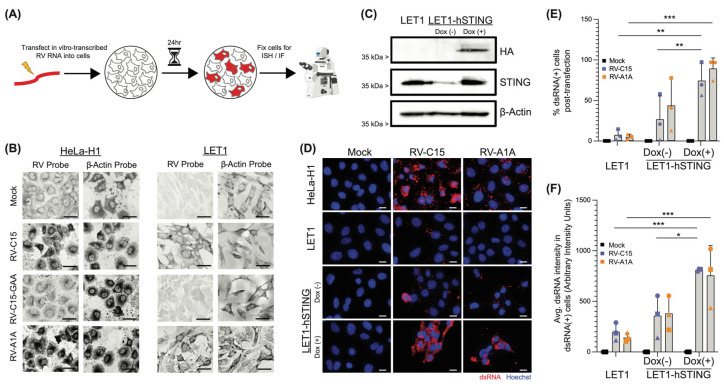
Mouse cells replicate RV-C, and replication is significantly increased upon expression of human STING. (**A**) Experimental design schematic. HeLa-H1 cells or LET1 cells were transfected in parallel with in vitro-transcribed RV RNA or mock-transfected. (**B**) In situ hybridization (ISH) probes (dark) identify the presence of negative-sense RV RNA, indicative of replicating RV for RV-C15-transfected LET1 (right) and HeLa-H1 cells (positive control, left), but not in mock or replication-deficient (RV-C15-GAA) genome-transfected cells at 24 hpt. Scale bar = 20 µm. (**C**) Western blot shows inducible expression of hSTING in modified LET1 cells. (**D**) Immunofluorescence-mediated detection of dsRNA (red) in HeLa-H1 cells (positive control), LET1, and LET1-hSTING with or without induction after transfection with either RV-C15 or RV-A1A RNA (positive control), but not in mock-transfected wells, confirms RV-C15 replication in a mouse cell background. Staining was performed 24 hpt. Nuclei (Hoechst; blue). Scale bar = 20 µm. (**E**) CellProfiler quantification of the % dsRNA-positive cells as from panel (**D**). Each individual point (square, circle, or triangle) represents the mean from an independent experiment. In each experiment, >30 cells were assayed across *n* = 4 immunofluorescence images taken at predetermined locations within the culture. Error bars represent standard deviation of the mean across experiments. (**F**) CellProfiler quantification of the average dsRNA intensity in dsRNA-positive cells. Statistical analysis in panels (**E**,**F**) was performed using a Mann–Whitney U test (two-tailed; 0.95% confidence interval; * *p* < 0.05, ** *p* < 0.01, *** *p* < 0.001).

We next sought to directly assess whether mouse cells are permissive for RV-C15 replication. To do so, we utilized murine LET1 cells and transfected in vitro-transcribed viral RNA to bypass the initial stages of infection. Notably, RV-A1A is unique in its ability to replicate in mice without prior adaptation [43]; thus, we utilized this RV as a positive control. We then visualized ongoing viral replication following mock-transfection, or transfection of RV-A1A or RV-C15 genomic RNA, using either a probe specific for a conserved region within the RV 5′-UTR of the anti-genome, or an antibody that detects dsRNA (J2; Figure 2A). HeLa-H1 cells were transfected in parallel as an additional control, and all cells were fixed 24 h later. Subsequent staining demonstrated in situ hybridization of the RV anti-genome probe in HeLa-H1 cells transfected with either RV-A1A or -C15 RNA, indicating replication of both RVs tested, with RV-A1A exhibiting robust signal (Figure 2B). RV-A1A- and RV-C15-, but not mock-transfected LET1 cells, also reacted with the RV 5’UTR probe, indicating that, indeed, these murine cells support RV-C15 replication. The addition of a replication-deficient RV-C15 virus (RV-C15-GAA) harboring mutations at the conserved GDD motif within the RNA-dependent RNA polymerase [44] further validated these findings, while hybridization of a control probe targeting the cellular-expressed β-actin transcripts in both cell types and under all conditions confirmed the RNA integrity in the samples.

Recently, McKnight et al. identified hSTING as a proviral factor that was able to augment RV-C replication [34]. Importantly, murine STING was unable to convey the same proviral effect, unless a murine-adapted virus (RV-A16L) was used [34]. Therefore, we assayed whether expression of hSTING in mouse cells would enhance replication of WT, non-mouse-adapted, RV-C. Towards this, we generated a LET1 cell line expressing an HA-tagged, tetracycline-inducible hSTING construct by stable transfection and subsequent puromycin selection. Upon induction with doxycycline, we observed an increase in hSTING expression, indicated by blotting for the HA-tag in cell lysates (Figure 2C). Then, to assess the impact of hSTING in these cells during RV replication, we followed the workflow detailed in Figure 2A, probing for replication using an antibody specific for dsRNA (Figure 2D and Supplemental Figure S1). As anticipated, HeLa-H1 cells supported replication of both RV-C15 and RV-A1A, as indicated by visualization of dsRNA-positive puncta in RV- but not mock-transfected conditions. Similarly, we observed the presence of dsRNA in LET1 cells and LET1 cells expressing hSTING with or without doxycycline induction after transfection with RV-C15 or RV-A1A, but not in mock-transfected cells. Although the levels of RV-C15 replication in LET1 cells appeared less robust following immunofluorescence- versus in situ hybridization-based assays, these assays target different products of ongoing RV replication and are, therefore, complementary, with both sets of results indicating that RV-C15 is capable of replicating in a mouse cell background. To quantify these data, we designed a custom CellProfiler [37] pipeline to assign dsRNA signal to a specific cell, and determined the percentage of dsRNA-positive cells (Figure 2E). We noted a statistically significant increase in the frequency of dsRNA+ cells in LET1-hSTING-expressing cultures upon transfection with RV-C15. Under these conditions, a vast majority of RV-C15-transfected, and nearly all RV-A1A-transfected, cells were replicating virus, suggesting comparable replication efficiency for these two RVs in a mouse cell background without further adaptation. The increased frequency of dsRNA+ cells in the LET1-hSTING condition could be attributed to differences in transfection efficiency, or the extent of viral replication in these cells, which may facilitate detection of dsRNA. Thus, we quantified the intensity of dsRNA signal in dsRNA+ cells and observed that, on a per-cell basis, the fluorescence intensity in RV-C15-transfected cells was higher in LET1-hSTING-expressing cells compared to uninduced LET1-hSTING cells, or the parental LET1 cell line (Figure 2F). 

### 3.3. Murine Cells Support the Production of Infectious RV-C Particles 

**Figure 3:**
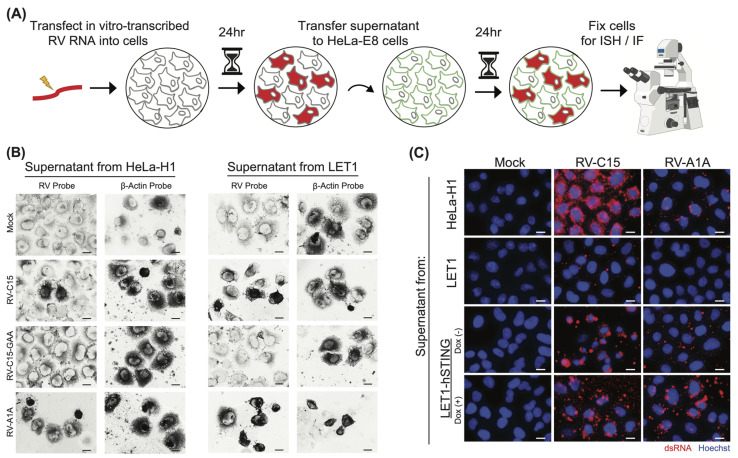
RV-C produced in mouse cells is infectious and capable of reinfection. (**A**) Experimental design schematic. HeLa-H1, LET1, or LET1-hSTING cells with or without induction of hSTING were transfected in parallel with in vitro-transcribed RV RNA or mock-transfected. At 24 hpt, supernatants were collected, filtered to remove any cellular material, and passaged to naïve HeLa-E8 cells. Infection of HeLa-E8 cells was confirmed by in situ hybridization or immunofluorescence staining. (**B**) In situ hybridization (ISH) probes (dark) identify the presence of negative-sense RV RNA, indicative of replicating RV in HeLa-E8 after inoculation with the supernatants described in (**A**), confirming infectious particle formation in LET1 cells. β-Actin ISH probes validate RNA integrity in the sample. Scale bar = 20 µm. (**C**) Immunofluorescence-mediated detection of dsRNA (red) in HeLa-E8 cells 24 hpi with supernatants harvested from HeLa-H1 (positive control), LET1, LET1-hSTING (with or without induction) cells previously transfected with either RV-C15 or RV-A1A RNA (positive control) but not mock. Nuclei (Hoechst; blue). Scale bar = 20 µm.

In the final stages of the viral life cycle, nascent virions are assembled and released from the infected cell. Since we found that LET1 cells are permissive for RV-C15 replication, we next designed an experiment, depicted in Figure 3A, to assess the production of infectious progeny. HeLa-H1 and LET1 cells were transfected in parallel as described in Figure 2A; however, in this case, the supernatants were collected 24 h post-transfection and then filtered to remove any potentially infected cells or virus-containing cellular debris. These supernatants were then transferred to naïve HeLa-E8 cells that had been previously modified to express hCDHR3, and shown to support the entire RV-C life cycle [33], and infected HeLa-E8 cells were identified using ISH probes targeting the RV anti-genome. Our results show that infectious particles were produced in HeLa cells after transfection with RV-C15 or RV-A1A RNA, but not in mock or replication-deficient RV-C15 conditions, as expected (Figure 3B). Importantly, we also detected RV probe reactivity in HeLa-E8 cells after inoculation with LET1 cell-derived supernatants originating from RV-A1A- or RV-C15-transfected cultures, confirming that mouse cells support the production of infectious RV particles.

To assess the impact of exogenous hSTING expression on infectious particle production, we also transfected RV genomes into HeLa-H1, LET1, and LET1-hSTING cells in the presence or absence of doxycycline prior to filtering and passaging the supernatants to naïve HeLa-E8 cells. The presence of infectious extracellular viral particles in the conditioned media from HeLa and LET1 cells was confirmed by staining HeLa-E8 cells inoculated with these media for replicating virus (dsRNA; Figure 3C and Supplemental Figure S2). We observed dsRNA signal in HeLa-E8 cells resulting from infection by virus produced in the HeLa-H1 control cells, but also from virus produced in LET1 cells alone and in the presence or absence of hSTING. 

### 3.4. CDHR3 KO Is Embryonically Lethal in Mice

Our work above suggested a potential incompatibility between RV-C and mCDHR3, and indicated that hSTING expression in mouse cells can increase replication. Therefore, we sought to generate mice expressing both of these human proteins to assess the impact on RV-C replication in vivo. Initially, we attempted to replace mCDHR3 entirely with the human ortholog. Towards this goal, mice heterozygous for constitutive knockout (KO) of mCDHR3 were generated by crossing animals containing loxP sites flanking exons 2 and 4 of mCDHR3 (C57BL/6NTac-Cdhr3-<tm6330Tac_A-C05>) with the ubiquitous Cre deleter model 12524 (experimental cross 1) from Taconic Biosciences. Resulting animals that were mCDHR3^+/−^ were then crossed, to determine if homozygous mCDHR3^−/−^ animals could be produced (experimental cross 2). However, the genotyping results (Table 3) of the 71 offspring revealed that none of the mice were mCDHR3^-/-^, thereby indicating that homozygous KO of CDHR3 is lethal. 

The production of mCDHR3^+/−^ and mCDHR3^+/+^ offspring also supports the conclusion of complete penetrance in homozygous lethality (Table 4). Given the expected 1:2:1 monohybrid genotypic ratio, we calculated an χ^2^ value of 8.34002 × 10^-06^ for the resulting genotypes of observed mouse pups. 

Additionally, to understand the inability to generate homozygous mCDHR3^−/−^ mice, we tracked pre-wean mortality over the course of a year (Table 5). Genotypic analysis revealed that the number of pups found dead or missing was very similar between experimental crosses 1 and 2, indicating that the lethality from CDHR3 KO is most likely embryonic. Notably, the litter size was slightly lower in experimental cross 2 compared to experimental cross 1.

### 3.5. AAV-Mediated Delivery of hSTING to hCDHR3 Transgenic Mice Promotes RV-C15 Replication In Vivo

**Figure 4:**
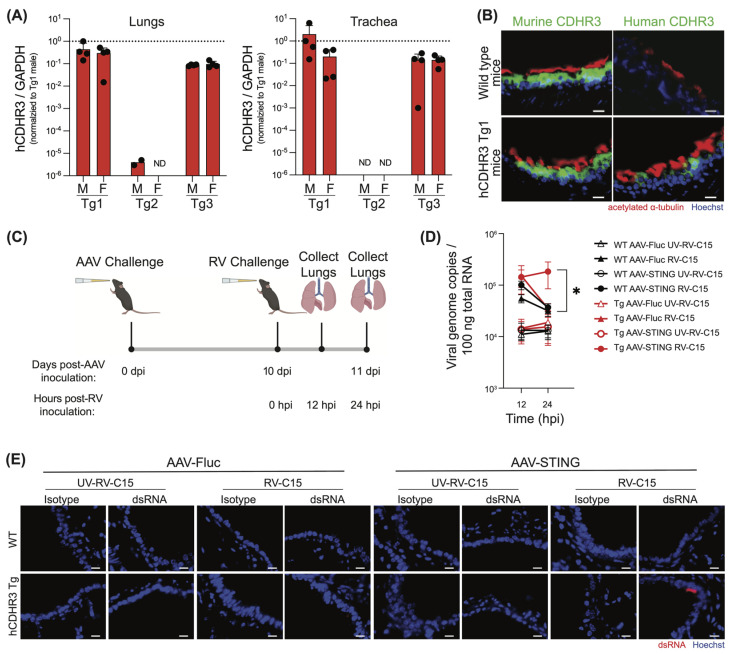
RV-C infection of transgenic mice expressing CDHR3 with the addition of human STING results in prolonged viral replication. (**A**) Human CDHR3 expression in the lungs and trachea of transgenic founder lines, Tg1, Tg2, and Tg3 by qPCR across *n* = 4 male (M) and *n* = 4 female (F) mice. Mean +/− standard deviation. ND = not detected. (**B**) Immunohistochemical detection of human and murine CDHR3 (green) in Tg1 mouse lung tissue compared to WT mice. Nuclei (Hoechst; blue) and cilia (acetylated alpha tubulin; red). Scale bar = 20 µm. (**C**) Experimental design schematic created with BioRender.com. Transgenic mice expressing human CDHR3 (Tg1) or WT mice were transduced with either human STING, or firefly luciferase (Fluc) via adeno-associated virus (AAV) by intranasal inoculation. On day 10 post-AAV transduction, mice were inoculated intranasally with RV-C15 or UV-inactivated RV-C15. Mice were then sacrificed, and total lungs were collected at 12 and 24 hpi. (**D**) qPCR detection of RV genome copy numbers at 12 and 24 hpi. Each point represents mean genome copy number from *n* = 11–14 mice assayed across three independent experiments. Black shapes represent WT mice and red shapes represent hCDHR3 Tg mice. Triangles represent mice receiving AAV-Fluc and circles represent mice receiving AAV-STING. Empty shapes represent UV-inactivated RV-C15 conditions while filled-in shapes represent mice receiving infectious RV-C15. Statistical analysis was performed using a Mann–Whitney U test (two-tailed; 0.95% confidence interval; * *p* < 0.05). (**E**) Immunohistochemical detection of dsRNA (J2; red) in paraffin-embedded mouse lung sections 24 hpi. Nuclei (Hoechst; blue). Scale bar = 20 μm.

Since homozygous mCDHR3 KO was embryonically lethal, we instead developed a transgenic C57BL/6 mouse that expressed hCDHR3 in addition to the endogenous mCDHR3 by random transgene integration. Because hCDHR3 was integrated at random using the endogenous promoter, it was necessary to assess these mice for their hCDHR3 expression profiles. Thus, we performed qPCR in the lungs and trachea from three hCDHR3 transgenic (Tg) lines created in parallel (Tg1, Tg2, and Tg3), including both male and female mice in our analysis to confirm that transgene integration did not occur in a sex-linked manner (Figure 4A). Notably, while hCDHR3 was only detected in the lungs of two Tg2 male mice, we found robust transgene expression in the lungs and trachea of both Tg1 and Tg3 mice. We then further compared the expression of hCDHR3 across tissues, including the upper gastrointestinal tract, spleen, kidney, liver, brain, and reproductive tissues (epididymis or ovaries), to yield a more complete understanding of the transgene expression profile in these mice (Supplemental Figure S3), and performed immunohistochemical staining for CDHR3 orthologs in lung tissue sections. As expected, we detected mCDHR3 in both WT and transgenic mice, while hCDHR3 was only observed in the transgenic lines, where it localized to the ciliated airway epithelium (Figure 4B and Supplemental Figure S4). Overall, the hCDHR3 expression profile was similar in Tg1 and Tg3 mice; however, Tg1 had higher mean expression in the lungs; therefore, we selected Tg1 for subsequent RV-C mouse challenge studies.

Having demonstrated the benefit of hSTING expression for RV-C replication in mouse cells in vitro, we also sought to determine if hSTING would have similar effects on infection in mice in vivo. Here, we devised a strategy to use an adeno-associated virus 6.2 vector to mediate the delivery of hSTING to the mouse respiratory tract. Initial experiments were aimed at determining the optimal temporal window for observing transgene expression in our model. Here, mice were intranasally inoculated with either AAV6.2-expressing firefly luciferase (AAV-Fluc) or PBS as a control, and subsequently euthanized on days 3, 6, 11, 18, 25, and 32 post-AAV inoculation (Supplemental Figure S5A). Nasal turbinates (Supplemental Figure S5B) and lungs (Supplemental Figure S5D) were collected, and flushed or inflated, respectively, with luciferin immediately prior to visualizing luciferase activity using an IVIS imager. Quantification of the luminescence signal in the nasal turbinates and lungs over the duration of this 32-day study revealed very low and consistent luciferase signal in the murine nasal turbinates across all time points (Supplemental Figure S5C) and a significant increase in luciferase signal in the murine lungs between day 3 and 6 post-AAV transduction (Supplemental Figure S5E). Since transgene expression in the lungs plateaued after day 6, we selected a time point after this (day 10) for the RV challenge in subsequent experiments. 

To next assess the impacts of hCDHR3 and hSTING expression alone, and in combination, on RV-C infection in mice, we inoculated WT and hCDHR3 transgenic (Tg1) mice intranasally with AAV expressing either hSTING (AAV-hSTING) or AAV-Fluc. We then challenged these mice on day 10 post-transduction with RV-C15, or UV-inactivated RV-C15, and harvested lungs at 12 and 24 hpi (Figure 4C). 

As anticipated, there were no significant changes in either weight (Supplemental Figure S6A) or health status (Supplemental Figure S6B) between experimental groups within this 24 hr time frame. We then extracted total RNA from the mouse lungs, and determined viral titers by quantifying genome copy numbers by qPCR (Figure 4D). In alignment with a prior report [32], RV-C15 titers decreased between time points in wild-type mice. In fact, we observed a loss in RV-C15 genome copies between 12 and 24 hpi in all conditions except in mice expressing both hCDHR3 and hSTING, where the mean RV-C titer was elevated in 10 out of 13 mice, and was found to be significantly higher than in all control conditions at the 24 hr time point. Thus, only hCDHR3 transgenic mice transduced with AAV-hSTING were able to sustain RV-C replication. To further validate this finding, we probed fixed mouse lung sections from this study for dsRNA using standard immunohistochemical protocols (Figure 4E) While the frequency of dsRNA-positive cells was low, this was not unexpected given our previous experience in staining for dsRNA following RV-C15 infection in human airway epithelial cultures that indicated a narrow time-frame for robust dsRNA detection [21]. Furthermore, we were able to identify replicating RV-C15 in mouse lungs from hCDHR3 transgenic mice that received AAV-hSTING at 24 hpi, but not in any other mouse lung tissues. 

## 4. Discussion

RV is a leading cause of mild upper respiratory tract infections [2,5,6,7,8]. Furthermore, the heightened risk for the development of asthma in children after infection by RV, and especially RV-C [8,45,46], highlights the importance of studying these pathogens. Human airway epithelial cultures provide a physiologically relevant platform to interrogate viral replication requirements and innate host responses and have been used to study RV-C in vitro [17,18,19,21]. However, they lack the ability to model adaptive immune responses and cannot be used to interrogate complex mechanisms driving virus-induced illness. RV tropism is limited to higher primates, and outside of a recent example of zoonotic spillover into chimpanzees, no natural RV infections have been documented in other species [26]. Therefore, given the limited utility of in vitro models for pathogenesis studies, the restricted host range of RVs, and the potential value of an in vivo mouse model of RV infection, we sought to understand the natural barriers to infection of mice. Examples of RV adaptations to allow for infection of mice or mouse cells have been previously described for major- and minor-group RVs [27,28,29,31,47,48,49,50,51]. Notably, RV-C infection was recently reported in 8-12-week-old female BALB/c mice and six-day-old C57BL/6J mice without any prior adaptation of virus or host [32,52]. While these data are challenging to explain in light of our own results, differences in mouse age and sub-strain, as well as in inoculum dose, could potentially contribute. Additional studies, ideally involving parallel inoculation of these RV-C mouse models alongside our model with the same virus stock, would be useful to resolve these discrepancies. Still, it is noteworthy that RV-C titers in these previously reported models progressively decline over time. 

Prior research into RV-C has primarily focused on CDHR3, which promotes viral entry into the airway epithelium, and has demonstrated that CDHR3 is expressed in ciliated cells across multiple tissues [38,39,53]. While we demonstrate a similar tissue expression profile for mCDHR3, including localization to the ciliated airway epithelium, a lack of homology at the amino acid level, including across EC1 and at residue 529, is in stark contrast to the high degree of conservation between hCDHR3 and chimpanzee CDHR3, a species where natural RV-C infection has occurred [26].

In analyzing the interaction of RV-C15 with the EC1 of CDHR3, we identified eight residues that differ between human and mouse CDHR3 orthologs. Among these differences, the N66R and I100A substitutions may have the greatest impact on virus–receptor binding by disrupting direct interactions between the two proteins. N66 makes polar interactions with G203 and T204 of VP2 in RV-C15 and is also adjacent to a salt bridge formed between two charged residues in RV-C15 and EC1 (R234 in RV-C15 VP2 and D102 in EC1). A substitution to a positively charged arginine at this site would not only disrupt these polar interactions but could also affect the formation of the adjacent salt bridge. Likewise, I100 forms several hydrophobic interactions with residues in RV-C15 (V73, P232, R234, and P235 of VP3), which would be lost by substitution to the much smaller alanine amino acid. The remaining six differing residues are on the periphery of the interface, but may still have a negative effect on virus–receptor binding. For the N99K substitution, there may not be room to accommodate the introduction of the much larger lysine residue. The G29S and S65P substitutions could have subtle effects on the backbone structure and, therefore, may have a negative impact on the orientation of the neighboring interfacial residues. While the I23L and L116V substitutions are relatively conserved, they may also have an impact on the conformation of the RV-C15—EC1 interface. The L24H substitution is also notable; however, Sun et al. showed that a similarly extreme L24D mutation had a minimal effect on the ability of hCDHR3 EC1 to either bind RV-C15 or support infection [41]. Therefore, it is possible that the L24H substitution may not negatively impact the RV-C15—EC1 interaction. In sum, when taken together, the totality of substitutions at and surrounding the predicted RV-C15—mCDHR3 EC1 interface are likely to destabilize RV-C—mCDHR3 interactions.

In humans, a non-synonymous single nucleotide polymorphism (SNP; rs6967330[A]) in CDHR3 results in an amino acid change at position 529 [42]. A cysteine at this position has been hypothesized to disrupt an expected disulfide bridge between C592 and C566, and, therefore, the presence of a tyrosine is thought to enhance protein stability. In line with this, SNP rs6967330 is associated with an increased risk for childhood development of asthma [42], and the presence of tyrosine correlates with increased CDHR3 expression at the cell surface [13,42]. Further, the C529Y mutation in CDHR3 is associated with increased RV-C binding in HeLa cells [40,54], as well as a heightened risk of respiratory tract illness with RV-C, but not other viruses [16]. Chimpanzees similarly harbor a tyrosine at this position, correlating with their natural susceptibility to RV-C infection. Mice, however, express a histidine at this position. Although the impact of CDHR3-H529 on surface expression and protein stability has yet to be empirically tested, the lack of a potentially disruptive cysteine here suggests that CDHR3-H529 is surface-stable. 

In probing the later stages of the viral life cycle, after entry and uncoating, we found that mouse cells support RV-C15 replication and the production of infectious progeny. These data closely mirror prior work that shows RV-1B and RV-A16 were capable of (limited) replication in mice without viral adaptation, provided they were able to gain entry into target epithelial cells [27]. Notably, hSTING, but not mSTING, was shown to support RV replication in human cells in vitro [34]. These results are not unexpected given that human and mouse STING share only 69% identity at the amino acid level. Expression of mouse STING was able to boost replication of a mouse-adapted variant (RV-A16L) harboring two mutations in the viral 2C protein [31,34]; however, co-immunoprecipitation studies to date have failed to demonstrate an interaction between hSTING and any RV protein [34]. Our work demonstrates that the expression of hSTING in mouse cells enhances RV-C15 and RV-A1A replication. While our Western blot data indicate that HA-tagged hSTING is undetectable in LET1-hSTING in the absence of doxycycline induction, we did note an increased percentage of dsRNA-positive cells and increased dsRNA intensity within this cell population compared to unmodified LET1 cells. These data suggest that either the inducible hSTING construct is leaky at baseline, or that through the process of selecting cells containing the hSTING construct, we simultaneously isolated a sub-population of LET1 cells that are naturally more permissive for RV replication. Nonetheless, these data together indicate that once RV-C15 genomes successfully reach the cytoplasm, the entirety of the RV-C life cycle can be completed, and that the expression of hSTING further increases levels of intracellular RV replication. Still, the mechanism underlying STING’s proviral function remains unknown, and these data further emphasize the need for additional research to ascertain the role of STING during RV replication. 

Towards our goal of generating a robust mouse model for RV-C, we initially attempted to develop a CDHR3 KO mouse line such that hCDHR3 would replace endogenous mCDHR3. Our discovery that the KO of mCDHR3 in mice is embryonically lethal was unanticipated, as there are very few data regarding CDHR3′s canonical functionality or contribution to adverse phenotypes. Indeed, CRISPR-Cas9 technology was previously used to knockout CDHR3 in primary human airway epithelial cultures, where it was reported that CDHR3 KO cells proliferate normally and give rise to ciliated cells during differentiation [55].

Using mice expressing hCDHR3 in addition to the endogenous mCDHR3, that were further supplemented with AAV-hSTING, we were able to detect dsRNA-positive epithelial cells and RV RNA levels at 24 hpi that were significantly higher than all other groups. These data indicate that expression of both hCDHR3 and hSTING are beneficial in this model. Still, how quickly RV-C15 is cleared remains unknown. Indeed, it is possible that other host factors must be supplied or humanized—or alternatively, depleted—in order to achieve a more robust, or more prolonged, infection. In human cells, RV prevents global host translation by 2A protease-mediated cleavage of eIF4G [56,57], while antiviral innate immune sensors of RV infections (e.g., RIG-I, MDA-5, and MAVS) are also targets of viral protease activity [58,59,60,61]. Notably, there are numerous instances of positive-sense RNA viruses (e.g., hepatitis A virus [62]; dengue virus [63,64]; Zika virus [65,66]) that fail to antagonize host immunity in mice and are, thereby, restricted for replication in this host. Further, we specifically note that the MAVS cleavage site targeted by RV-C is not conserved in mice, with a critical change at P1′ [61]. Therefore, it is possible that RV-C15 is not able to adequately control the murine innate immune response, and we hypothesize that these transgenic mice depleted for innate immune sensors or downstream effector molecules may result in prolonged RV-C15 infection. 

Ultimately, this study characterizes the role of hSTING during RV-C15 infection in mouse cells in vitro and in vivo. Our data represent the initial blueprint towards generating a mouse model of RV-C infection, utilizing transgenic mice expressing hCDHR3 and AAV-mediated delivery of hSTING. We speculate that hCDHR3 expression facilitates uptake of RV-C15 and hSTING expression enables faster replication, which allows RV-C to overcome the murine immune system during initial RV-C replication. Alternatively, hSTING expression may impede normal immune signaling in the mouse—thereby explaining the observed differences in RV-C15 titers at 24 hpi. Future studies should assess other RV-C serotypes and characterize both viral replication and host response over a longer time course. While these mice likely do not encompass the totality of factors and conditions needed, they suggest that an RV-C mouse model is ultimately achievable. 

## Figures and Tables

**Table 1:** Primers used in determining the presence of the hCDHR3 transgene.

Primer Name	Sequence	Expected Fragment Size (Base Pairs)
Control FWD	5’-GAGACTCTGGCTACTCATCC-3’	585
Control REV	5’-CCTTCAGCAAGAGCTGGGGAC-3’	
5’-end FWD	5’-CCTTGTTCATGCAGCACTTG-3’	307
5’-end REV	5’-CCCAAACCCGACAGTTGTC-3’	
Exon 1 FWD	5’-TACCGCCAAAGCCTCTGT-3’	306
Exon 1 REV	5’-CTCAATTGAACAAGCCTCCC-3’	
Exon 6 FWD	5’-TATGCACTTTCCAGAGATCTTGTG-3’	500
Exon 6 REV	5’-CCTTGTATTAAATGTGCCGGAG-3’	
Exon 12 FWD	5’-CCATGGCAGTTAGGACATCTG-3’	369
Exon 12 REV	5’-AGAGACCCTAAGTCAACCAGTTG-3’	
3’-UTR FWD	5’-CTGGGGTTCTCATTGTTTGC-3’	310
3’-UTR REV	5’-CAGCTGGAACTAGAACTCTGTGG-3’	
3’-end FWD	5’-GCGCCTCACTTTGTCACC-3’	322
3’end REV	5’-GGCCTAGAATTCTCTGTGGG-3’	

**Table 2:** Primers used in confirming transgene homozygosity.

Primer Name	Sequence
CDHR3-Z.28721-FWD	5’-GATGATGACAGTGAGGCACCAA-3’
CDHR3-Z.28721-REV	5’-CGCTCCCCACTCCAGATG-3’
CDHR3-Z.28721-Probe	5’-6FAM-CAACAGATTCAACTTCAC-MGBNFQ-3’
BETA-ACTIN33-FWD	5’-CATGCAAGGAGTGCAAGAACA-3’
BETA-ACTIN95R-REV	5’-GGAGCCCCTGTCCTGAGACT-3’
VIC-BETA-ACTINZ2-Probe	5’-VIC-AGCTAAGTTCAGTGTGCTGG-MGBNFQ-3’

**Table 3:** Genetic crosses of Cre^+/+^ mice with mCDHR3^+/−^ mice are unable to produce homozygous mCDHR3^−/−^ offspring.

Cre Genotype	mCDHR3 Genotype	Total Number of Offspring
Cre^+/+^	mCDHR3^−/−^	0
Cre^+/+^	mCDHR3^+/−^	48
Cre^+/+^	mCDHR3^+/+^	23

**Table 4:** Genetic crosses of mCDHR3^+/-^ mice result in non-Mendelian offspring ratios, suggesting mCDHR3^−/−^ mice are non-viable.

Genotype	Expected Number of Offspring	Observed Number of Offspring	% of Total Offspring
mCDHR3^−/−^	18	0	0%
mCDHR3^+/−^	36	48	67.61%
mCDHR3^+/+^	18	23	32.39%

**Table 5:** Assessment of pre-wean mouse mortality suggests mCDHR3^−/−^ mice are embryonically lethal.

Experiment	Missing + Found Dead	Weaned Pups	Pre-Wean Mortality %	Average Litter Size at Weaning
Experimental Cross 1	24	121	16.55%	4.5%
Experimental Cross 2	18	94	16.07%	3.9%

## Data Availability

The original contributions presented in the study are included in the article/Supplementary Materials; further inquiries can be directed to the corresponding author.

## Supplementary Materials

The following supporting information can be downloaded at https://www.mdpi.com/article/10.3390/v16081282/s1, Figure S1: Human STING expression increases dsRNA detection in mouse cells; Figure S2: Supernatants derived from mouse cells with and without hSTING expression contain infectious viral particles capable of infecting HeLa-E8 cells; Figure S3: CDHR3 expression in transgenic mouse lines by qPCR; Figure S4: Immunohistochemical detection of CDHR3 protein in wild type and transgenic mouse airway epithelium; Figure S5: Intranasal delivery of AAV6.2 results in transgene expression in the lung; Figure S6: Wild type and hCDHR3 transgenic mice lack clinical signs of disease 24 hours after rhinovirus inoculation.
