# Supplementary material for: Human Stimulator of Interferon Genes Promotes Rhinovirus C Replication in Mouse Cells In Vitro and In Vivo
Source: Viruses. 2024 Aug 10;16(8):1282. doi: 10.3390/v16081282 (PMC11358906; doi:10.3390/v16081282)
Supplement: Supplementary file 1 [file viruses-16-01282-s001.zip › viruses-3017046-supplementary.pdf]

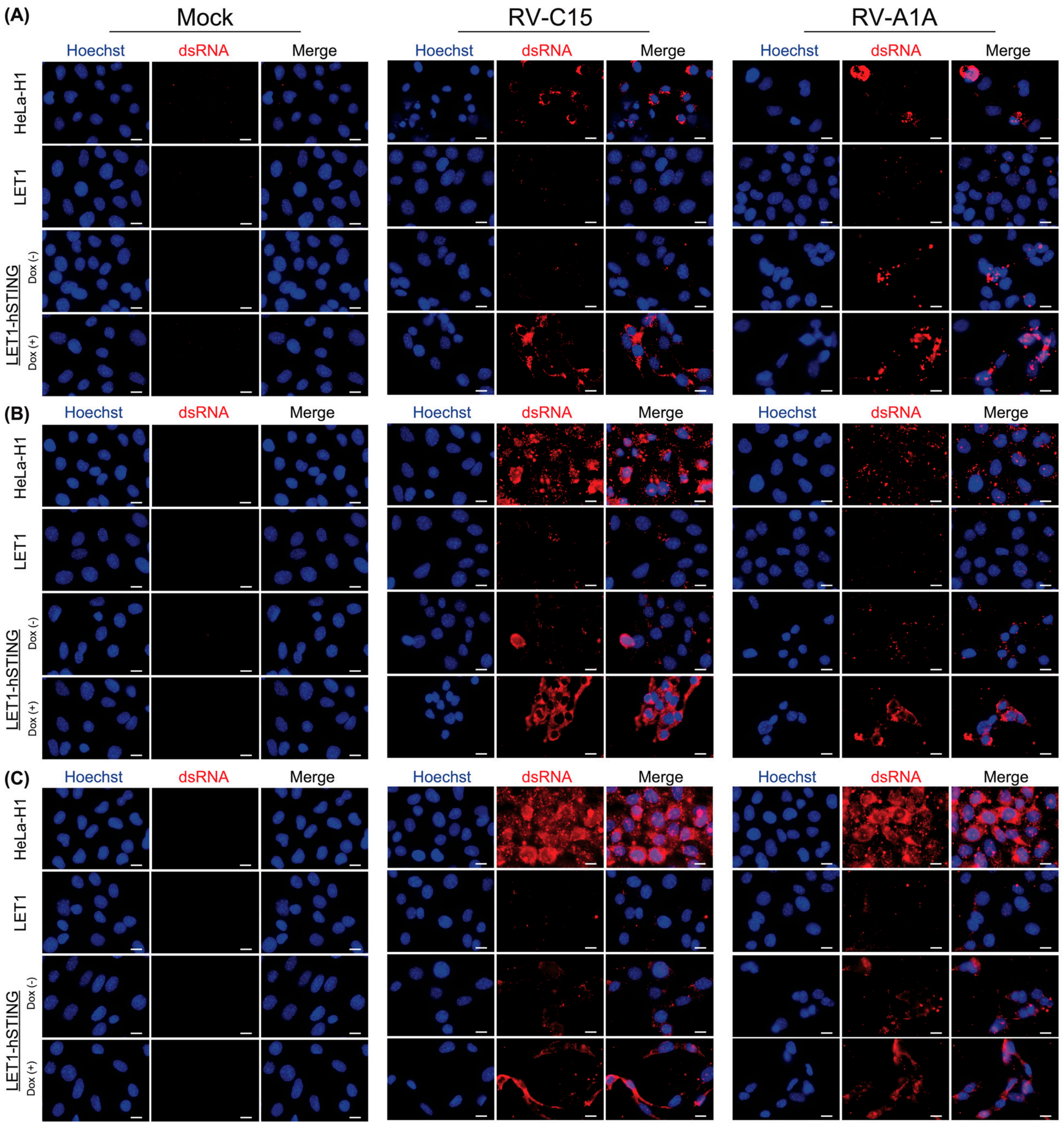

Supplemental Figure S1: Human STING expression increases dsRNA detection in mouse cells. Immunofluorescence-mediated detection of dsRNA (red) in HeLa cells (positive-control), LET1, and LET1-hSTING with or without induction after transfection with either RV-C15 or RV-A1A RNA (positive-control) but not in mock-transfected wells, confirms RV-C15 replication in a mouse cell background. Staining was performed 24 hpt from three separate biological replicates (**A**), (**B**) and (**C**). Nuclei (Hoechst; blue). Scale bar = 20 µm. Merged data shown in panel (B) are replicated in Figure 2D.

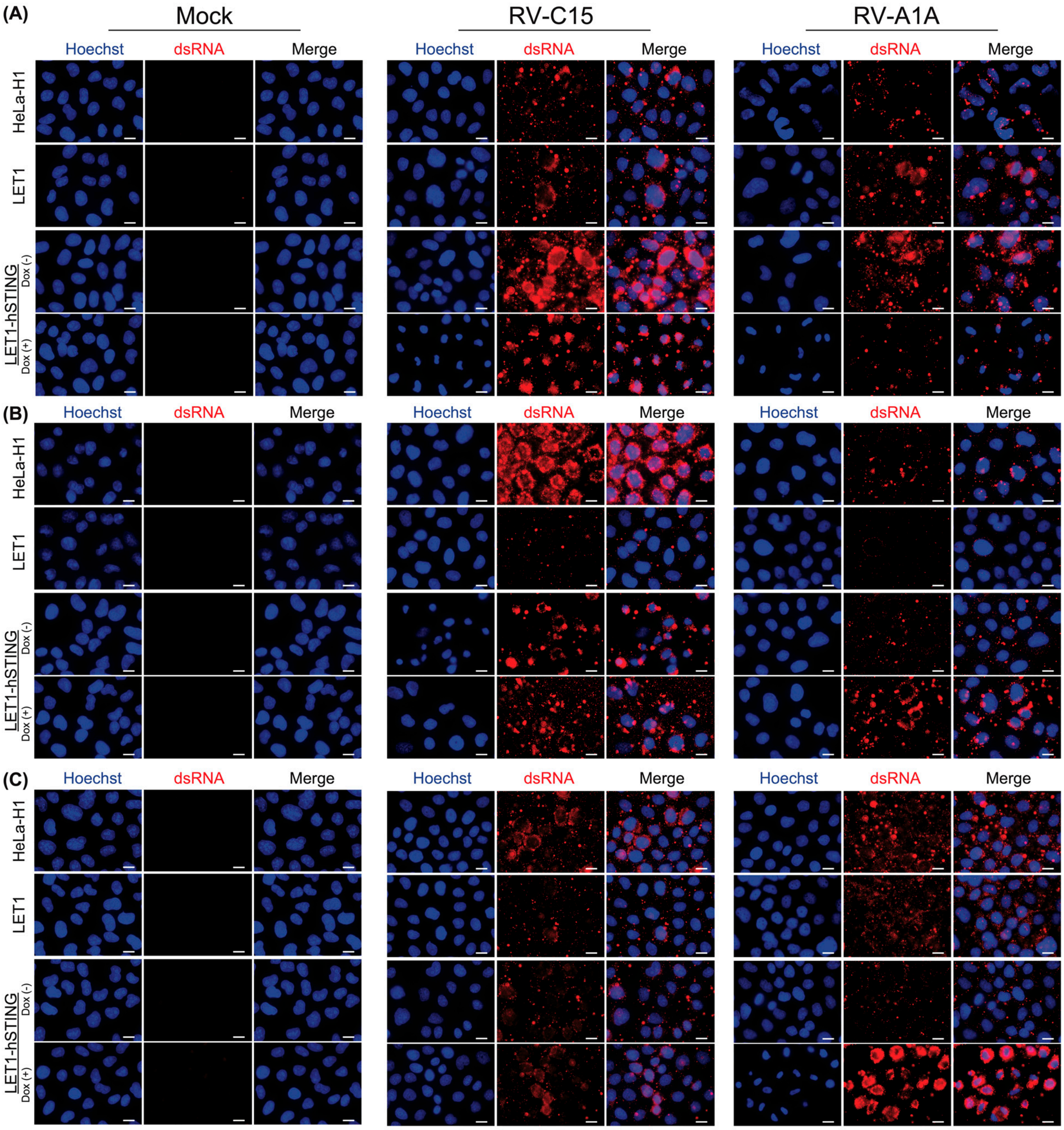

Supplemental Figure S2. Supernatants derived from mouse cells with and without hSTING expression contain infectious viral particles capable of infecting HeLa-E8 cells. Immunofluorescence-mediated detection of dsRNA (red) in HeLa-E8 cells 24 hpi with supernatants harvested from HeLa-H1 (positive-control), LET1, LET1-hSTING (with or without induction) cells previously transfected with either RV-C15 or RV-A1A RNA (positive-control) but not mock from three separate biological replicates (**A**), (**B**) and (**C**). Nuclei (Hoechst; blue). Scale bar = 20 μm. Merged data shown in panel (B) are replicated in Figure 3C.

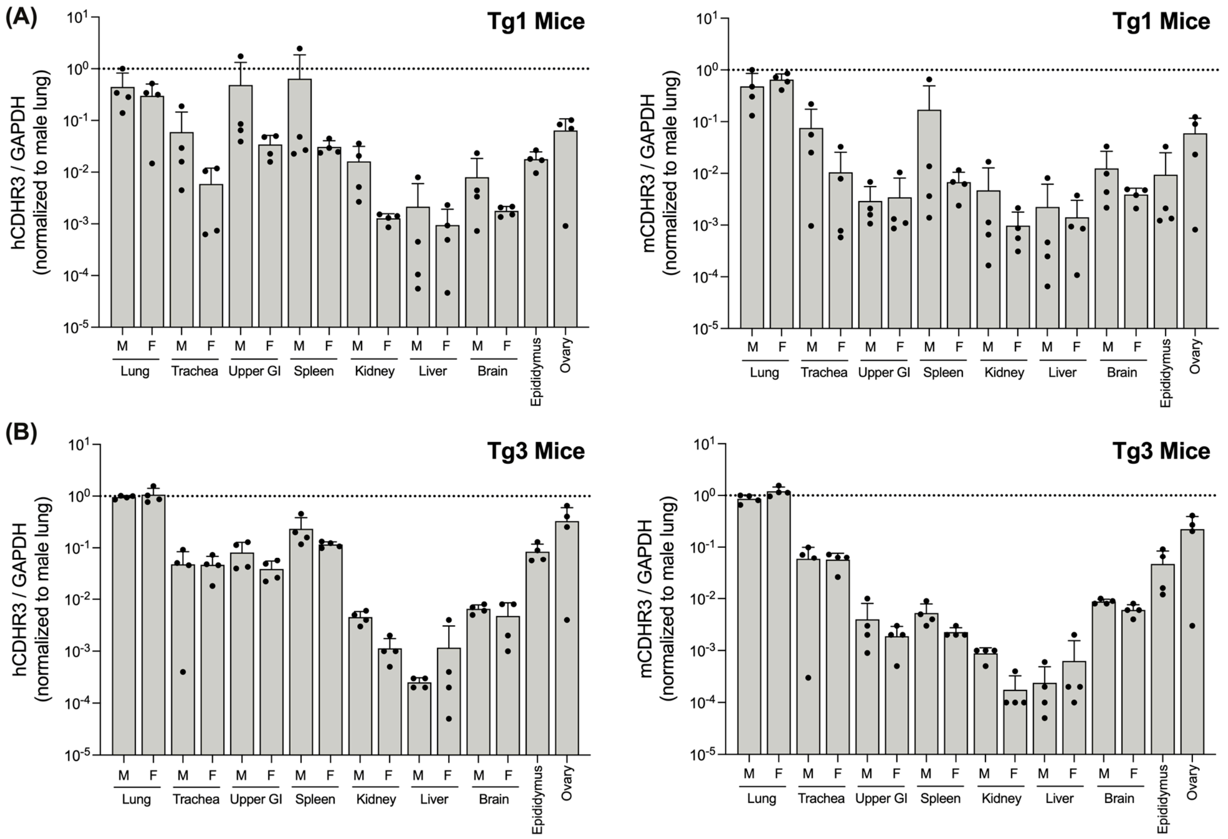

Supplemental Figure S3. CDHR3 expression in transgenic mouse lines by qPCR. hCDHR3 and mCDHR3 expression in male (M) and female (F) Tg1 (**A**) and Tg3 (**B**) mouse tissues by qPCR. Each dot represents an individual mouse. Bars represent mean +/- standard deviation.

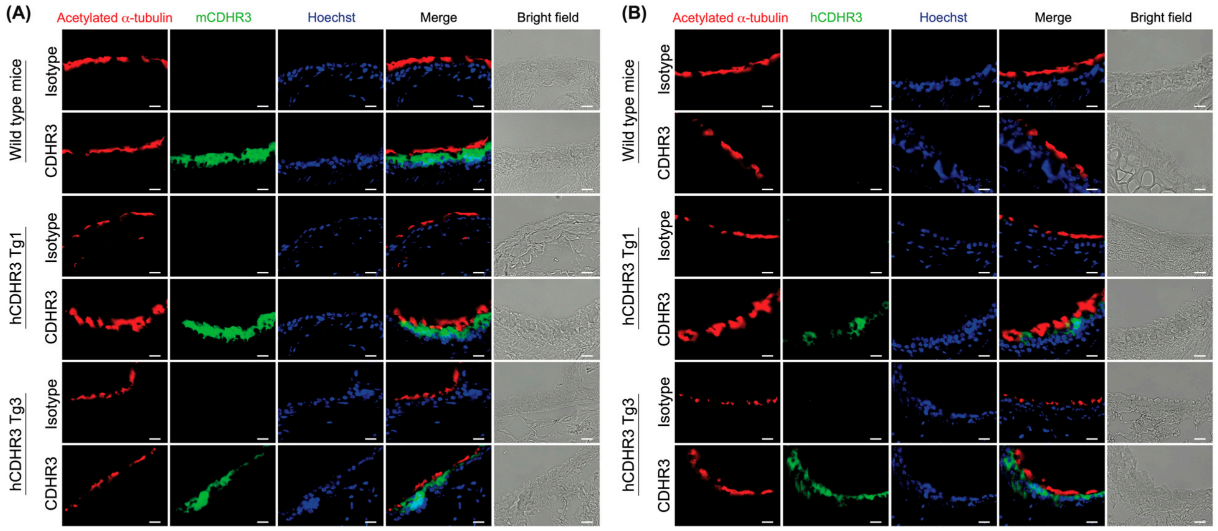

Supplemental Figure S4. Immunohistochemical detection of CDHR3 protein in wild type and transgenic mouse airway epithelium. Immunohistochemical analysis of mCDHR3 (green; **A**) and hCDHR3 (green; **B**) in WT, Tg1, and Tg3 mouse lung sections. Nuclei (Hoechst; blue) and cilia (acetylated-alpha tubulin; red). Scale bar = 20 μm. Merged data for WT and Tg1 mice are replicated in Figure 4B.

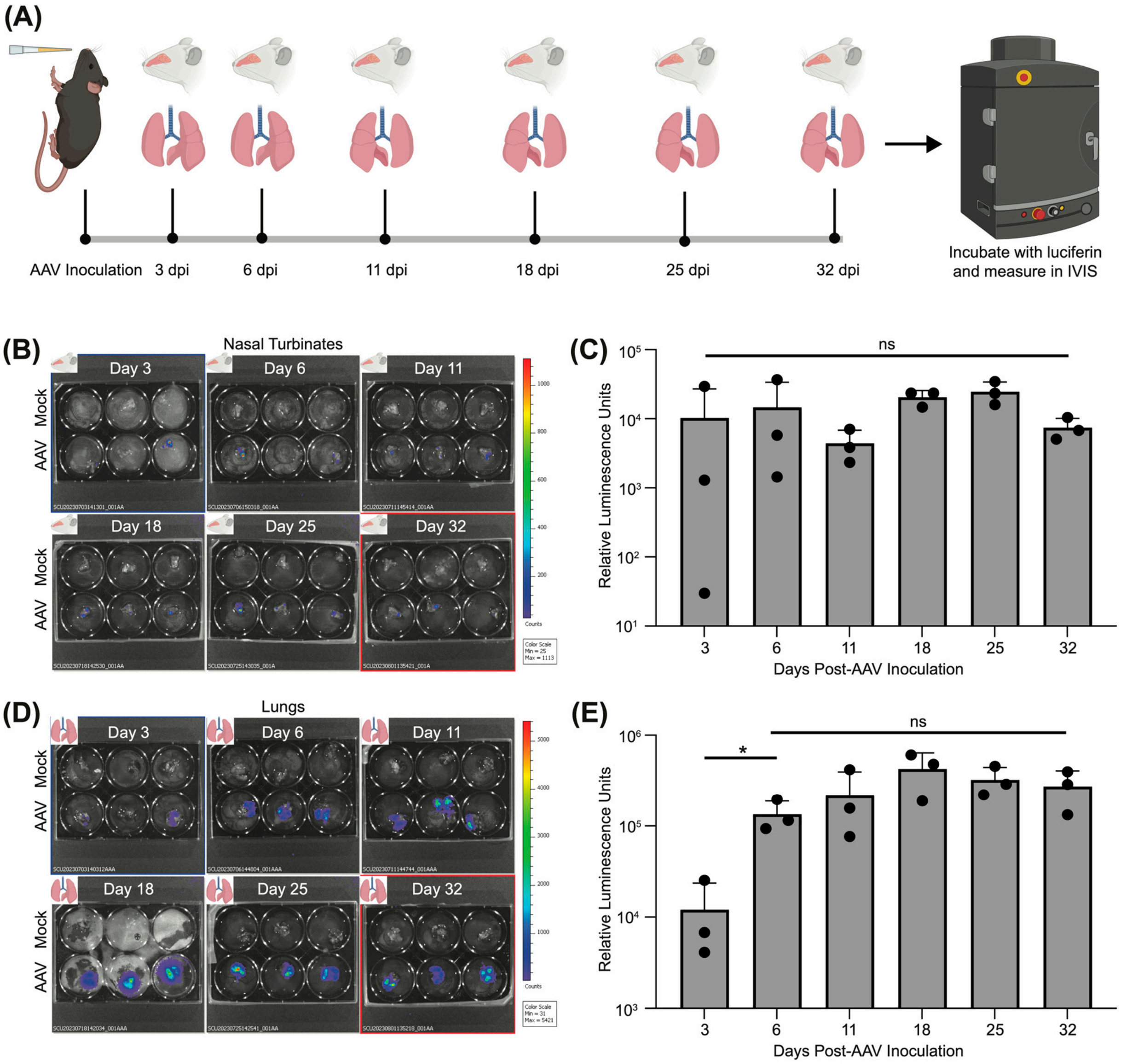

Supplemental Figure S5. Intranasal delivery of AAV6.2 results in transgene expression in the lung. (**A**) Experimental design schematic created with BioRender.com. N=36 mice were intranasally inoculated with either AAV-Fluc or PBS and lungs and nasal turbinates were assayed for luciferase activity 3, 6, 11, 18, 25, and 32 dpi. (**B**) IVIS images of nasal turbinates across timepoints. (**C**) Quantification of luciferase activity in nasal turbinates across timepoints. Mean +/- standard deviation. (**D**) IVIS images of the lungs across timepoints. (**E**) Quantification of luciferase activity in the lungs across timepoints. Mean +/- standard deviation. Statistical analysis in panels C and E was performed using an unpaired t-test; *p<0.05; ns = non-significant.

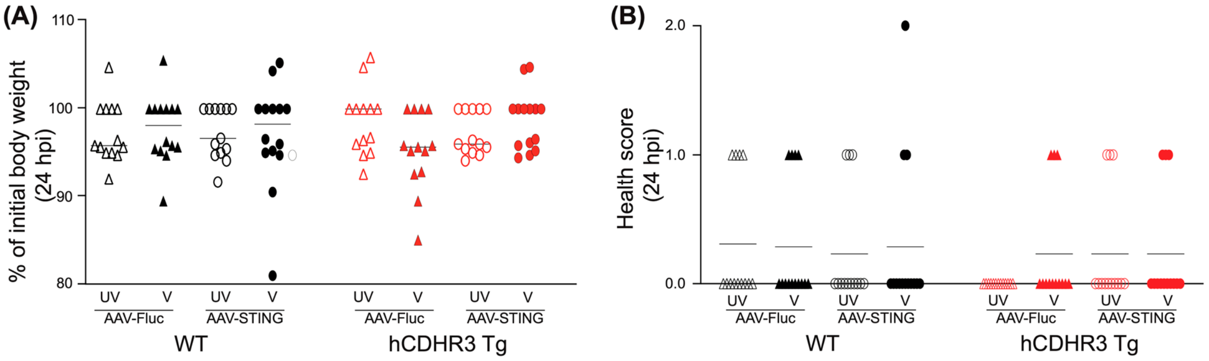

Supplemental Figure S6: Wild type and hCDHR3 transgenic mice lack clinical signs of disease 24 hours after rhinovirus inoculation. Mouse weights (**A**) and health scores (**B**) were assessed at T=24 hpi across WT (black) and Tg (red) mice that received either AAV-Fluc (triangles) or AAV-STING (circles) followed by either UV-inactivated RV-C15 (open shapes) or infectious RV-C15 (closed shapes).
